# Soluble interleukin-2 receptors in ulcerative colitis

**DOI:** 10.1155/S096293519300016X

**Published:** 1993

**Authors:** O. H. Nielsen, J. Brynskov

**Affiliations:** 1 Department of Medical Gastroenterology C Herlev Hospital University of Copenhagen Herlev Ringvej,Herlev DK-2730 Denmark; 2 Department of Medical Gastroenterology F Glostrup Hospital University of Copenhagen Denmark

## Abstract

T-Cell activation results in the release or shedding of a soluble form (45 kDa) of the cellular (55 kDa) low-affinity interleukin-2 receptor (α-chain) (slL-2R). The present study was performed to investigate if the serum concentration of sIL-2R is a marker of disease activity in patients with ulcerative colitis (UC), a chronic inflammatory bowel disease. Twenty-seven UC patients (about half of them in remission) and 13 healthy volunteers were studied, sIL-2R concentrations were measured by an enzyme-linked immunosorbent assay, and significantly elevated median sIL-2R values were found in clinically active UC (150 pg/ml; range 100–420), compared to inactive UC (145 pg/ml; range 110–255), and healthy controls (110 pg/ml; range 80–165) (p < 0.01). There was no correlation between sIL-2R concentrations and extent of the disease. Due to the overlap of serum sIL-2R concentrations between different disease stages and controls, the general diagnostic value seems to be limited. However, since slL-2R release is an IL-2 dependent phenomenon, we conclude that the demonstration of increased serum sIL-2R concentrations in UC suggests the existence of an enhanced T-cell activation *in vivo* in this disease. Further longitudinal studies are required to elucidate if repeated measurements of sIL-2R levels provide an additional way of monitoring UC disease activity in individual patients.


Mediators of Inflammation, 2, 115–118 (1993)

### Soluble interleukin-2 receptors in ulcerative colitis

O. H. Nielsen,[1,CA] and J. Brynskov[2]

[1]Department of Medical Gastroenterology C, Herlev Hospital, University of Copenhagen, Herlev Ringvej, DK-2730 Herlev, Denmark; [2]Department of Medical Gastroenterology F, Glostrup Hospital, University of Copenhagen, Denmark

[CA] Corresponding Author

**T-CELL activation results in the release or shedding of a soluble form (45 kDa) of the cellular (55 kDa) low-affinity interleukin-2 receptor (α-chain) (sIL-2R). The present study was performed to investigate if the serum concentration of sIL-2R is a marker of disease activity in patients with ulcerative colitis (UC), a chronic inflammatory bowel disease. Twenty-seven UC patients (about half of them in remission) and 13 healthy volunteers were studied. sIL-2R concentrations were measured by an enzyme-linked immunosorbent assay, and significantly elevated median sIL-2R values were found in clinically active UC (150 pg/ml; range 100–420), compared to inactive UC (145 pg/ml; range 110–255), and healthy controls (110 pg/ml; range 80–165) ($p < 0.01$). There was no correlation between sIL-2R concentrations and extent of the disease. Due to the overlap of serum sIL-2R concentrations between different disease stages and controls, the general diagnostic value seems to be limited. However, since sIL-2R release is an IL-2 dependent phenomenon, we conclude that the demonstration of increased serum sIL-2R concentrations in UC suggests the existence of an enhanced T-cell activation *in vivo* in this disease. Further longitudinal studies are required to elucidate if repeated measurements of sIL-2R levels provide an additional way of monitoring UC disease activity in individual patients.**

**Key words:** Inflammation, Interleukin-2 receptors, T lymphocytes, Ulcerative colitis

### Introduction

The etiology of ulcerative colitis (UC) is unknown, but there is increasing evidence that immunological factors, including T-cell mediated immune functions, play a pathogenetic role in inflammatory bowel disease (IBD).[1,2]

T-cell proliferation and differentiation depend primarily on the interplay between interleukin-2 (IL-2) and its specific receptor (IL-2R), which is expressed on activated, but not on resting T-cells.[3–5] The biologically active, high-affinity IL-2R complex consists of a high-affinity 75 kDa β-chain, and a low-affinity 55 kDa α-chain.[6] During activation, T-cells release a soluble (45 kDa) form of the low-affinity IL-2R α-chain (sIL-2R), which is detectable by enzyme-linked immunosorbent assay (ELISA) in serum and in inflamed gut tissue.[7–9] Circulating sIL-2R levels may be of potential clinical value for the disease activity assessment in IBD,[10] and may also play a role in the regulation of T-cell activation, because it binds free IL-2.[11]

The aim of the present study was to evaluate if serum concentration of sIL-2R is a marker of disease activity in patients with different activity stages of UC.

### Materials and Methods

*Patients:* Twenty-seven patients (17 women, ten men) (median age 40 years, range 19–77 years) with UC,[12] were included in the study from the IBD out-patient clinic of Herlev Hospital, together with 13 healthy volunteers (ten women and three men) (median age 34 years, range 25–49 years).

The disease duration (time from diagnosis to inclusion in the study) had a median of 6 years (range 1–22 years). Eighteen patients (67%) had distal (proctosigmoid) disease, and nine (33%) were more extensively affected.

Disease activity was assessed on an ordered scale from 1 (remission) to 4 (severe activity).[13] About one half of the patients ($n = 13$; 48%) were in remission, and about one half ($n = 14$; 52%) had moderately active disease (grade 3). Only two (14%) UC patients with active disease received drugs (sulphasalazine or 5-aminosalicylic acid). Consequently none received glucocorticoid or immunosuppressive treatment at the time of blood sample collection.

The study was approved by the Scientific Ethical Committee of the Copenhagen County. In accordance with the Second Helsinki Declaration,

© 1993 Rapid Communications of Oxford Ltd informed consent was obtained from all patients and healthy volunteers before entering the study.

*sIL-2R:* Serum sIL-2R concentrations were measured as described in detail previously[10] using a sandwich ELISA (T Cell Sciences, MA, USA). The ELISA incorporates a primary monoclonal antibody against the Tac-epitope,[14] and a second monoclonal antibody (7G7/B6), which binds to an IL-2R epitope (α-chain) different from that of anti-TAC and IL-2.[15] Units of sIL-2R were calculated from a standard curve. Previous studies have shown 1 ELISA unit to be equivalent to 0.3 pg purified receptor.[16] The coefficient of variation was less than 0.10.

*Statistics:* Variance analysis of unpaired data for trend was performed by the Jonckheere–Terpstra test, which is one-tailed; unpaired data by the Mann–Whitney test (two-sided). The significance limit for $p$ values was less than 5%.

### Results

The serum sIL-2R concentrations were significantly increased in UC patients (median 150 pg/ml, range 100–420) compared with controls (median 110 pg/ml, range 80–165) ($p < 0.02$).

As shown in Fig. 1, the median sIL-2R concentration was significantly higher in UC patients with active disease (150 pg/ml), compared with those in remission (145 pg/ml), and controls (110 pg/ml) ($p < 0.01$) according to a variance analysis.

There was no significant difference in sIL-2R concentrations between patients with proctosigmoiditis (median 140 pg/ml) and those with more extensive colitis (median 155 pg/ml) ($p = 0.19$), (Fig. 2).

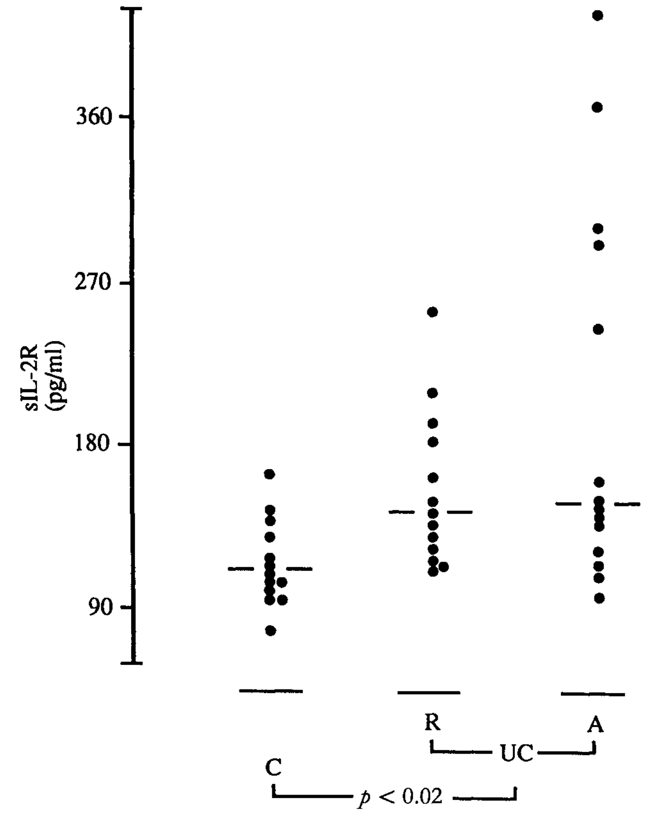

FIG. 1. Serum concentrations (pg/ml) of soluble interleukin-2 receptors (sIL-2R) in 27 patients with ulcerative colitis (UC) (14 with active disease (A), and 13 in remission (R)) and 13 healthy volunteers (C). Bars represent median values.

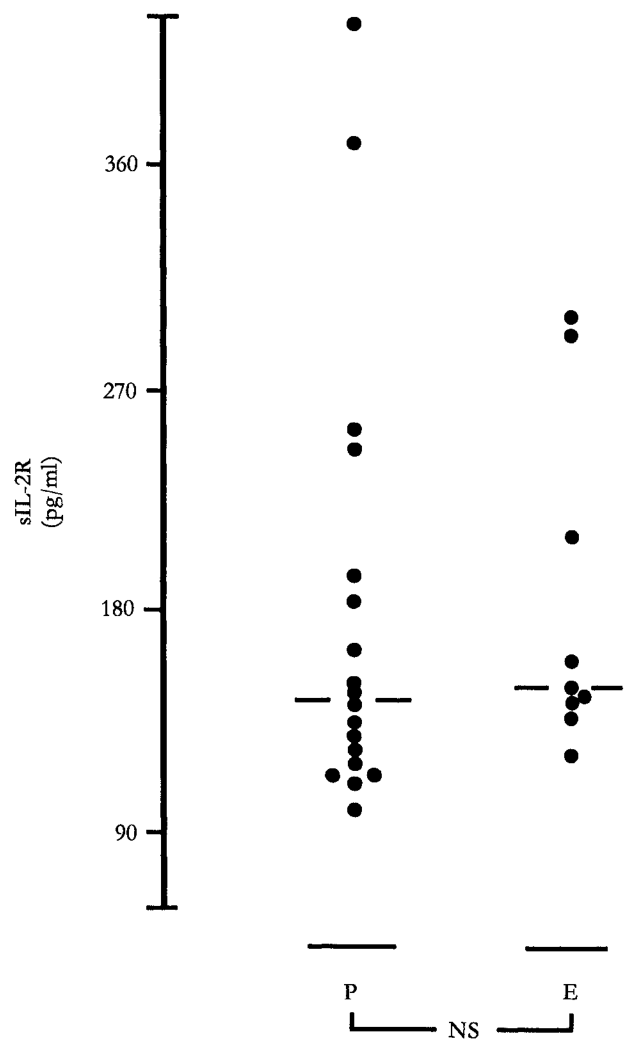

FIG. 2. Serum concentrations (pg/ml) of sIL-2R among 27 patients with ulcerative colitis (UC) (18 with proctosigmoiditis (P) and nine with a more extensive colitis (E)). Bars represent median values. (NS = not significant.)

### Discussion

IL-2 is a T-cell-derived cytokine which plays a key role in the regulation of T-lymphocyte functions.[1] Peripheral blood lymphocyte activation is a prominent feature of many putative autoimmune diseases,[17–19] and sIL-2R has been proposed as a simple method of assessing *in vivo* T- (and B-) cell activation.[20] For instance, serum sIL-2R concentrations correlate with disease activity in rheumatoid arthritis, atopic eczema, Behçets disease,[21–23] systemic lupus[24] and chronic autoimmune hepatitis.[25]

As regards IBD, there is an increased infiltration of lymphocytes in the inflamed lamina propria as compared to normal bowel.[2,26] Both peripheral and mucosal T-cells from patients with active IBD express IL-2R on their surfaces, whereas in the normal colon only a few lymphocytes stain with the antibody to the low-affinity IL-2R (Tac-antigen).[27]

A number of studies in patients with CD have shown a markedly accelerated production of sIL-2R by peripheral blood mononuclear cells which corresponds to the consistent demonstration of significantly increased serum sIL-2R levels in active, but not in inactive disease stages.[9,28–30] There seems, however, to be a differential expression of cellular IL-2R in patients with CD and UC[31] which has been proposed to distinguish between the two disorders. Thus, the IL-2R (CD25) bearing cells are abundant in the submucosa in CD but to a lesser extent in UC. Furthermore, the CD25+ cells in CD comprise 58–88% CD3+, CD4+, and CD8−, indicating that they are T-cells, whereas in UC the CD25+ cells comprise more than 80% CD3−, CD4+, and HLA-DR+, indicating that they are macrophages.[31] In two previous studies on circulating sIL-2R concentrations in patients with active UC, nearly all patients studied received either an oral glucocorticoid, azathioprine, sulphasalazine/5-aminosalicylic acid, or combinations thereof.[29,32] Although these drugs may interfere with different steps in the IL-2 dependent pathway of immune activation,[1,33] significantly higher serum levels of sIL-2R were found in patients with active UC, which is in accordance with the present findings, based on mainly untreated patients. Consequently, ongoing treatment with these drugs seems to have little influence on sIL-2R levels, provided that these patients have active disease.

Lamina propria lymphocytes from UC patients release significantly less amounts of sIL-2R *in vitro* as compared with controls.[32,34] This finding is somewhat difficult to reconcile with the repeated demonstration of increased sIL-2R levels in tissue homogenates from the same patient category,[19,29] but it illustrates that the precise cellular source of sIL-2R in UC is unknown. Apart from confirming elevated sIL-2R levels in patients with active and mainly untreated UC, the present study also shows that even UC patients in remission have significantly elevated serum sIL-2R levels.

There was no difference between circulating sIL-2R levels in patients with proctosigmoiditis and those with more extensive colitis, suggesting an equal degree of immune activation. This may be in accordance with the clinical experience that the intensity of immunosuppressive treatment should be chosen on the basis of the severity rather than the extent of the disease.[35]

The biological role of raised sIL-2R levels is yet unknown. sIL-2R may have an immunoregulatory role by competing with the IL-2R complex on cell surfaces for free IL-2, thus facilitating a down-regulation of the immune response.[11] Accordingly, when blood mononuclear cells from patients with active IBD are stimulated with IL-2 *in vitro*, the result is an increased cellular expression of low affinity IL-2R which, apart from binding free IL-2, also has been proposed to represent biologically active binding sites.[36]

The release of sIL-2R is an IL-2 dependent phenomenon.[37] We conclude that the demonstration of increased serum sIL-2R levels in patients with UC suggests an enhanced T-cell activation in this disorder. However, the overlaps between different disease stages, and between patients and controls, limit the general diagnostic value of this measure. Longitudinal studies are required to elucidate a possible role of serum sIL-2R as an additional marker of disease activity in individual patients who act as their own controls.

### References

1. Brynskov J, Nielsen OH, Ahnfelt-Rønne I, Bendtzen K. Cytokines in inflammatory bowel disease. *Scand J Gastroenterol* 1992; **27**: 897–906.
2. Jewell DP, Snook JA. Immunology of ulcerative colitis and Crohn's disease. In: Allan RN Keighley MRB, Alexander-Williams J, Hawkins CF, eds *Inflammatory bowel diseases*. 2nd edition. London: Churchill Livingstone, 1990: 127–146.
3. Trowbridge I. Interleukin-2 receptor proteins. *Nature* 1987; **327**: 461–462.
4. Cantreel DA, Smith KA. The interleukin-2 T-cell system. *Science* 1984; **224**: 1312–1316.
5. Wang HM, Smith KA. The interleukin 2 receptor: functional consequences of its bimolecular structure. *J Exp Med* 1987; **166**: 1055–1069.
6. Smith KA. The two-chain structure of high affinity IL-2 receptors. *Immunol Today* 1987; **8**: 8–11.
7. Rubin LA, Kurman CC, Fritz ME, Biddison WE, Boutin B, Yarchoan R, Nelson DL. Soluble interleukin 2 receptors are released from activated human lymphoid cells *in vitro*. *J Immunol* 1985; **135**: 3172–3177.
8. Nelson DL. Soluble interleukin-2 receptors: analysis in normal individuals and in certain disease states. *Fed Proc* 1986; **45**: 377.
9. Brynskov J, Tvede N, Vilien M, Andersen CB, Bentzen K. Increased concentrations of interleukin 1$\beta$, interleukin 2, and soluble interleukin-2 receptors in endoscopical mucosal biopsy specimens with active inflammatory bowel disease. *Gut* 1992: **33**: 55-58.
10. Brynskov J, Tvede N. Plasma interleukin-2 and a soluable/shed interleukin-2 receptor in serum of patients with Crohn's disease. Effect of cyclosporin. *Gut* 1990; **31**: 795-799.
11. Rubin LA, Jay G, Nelson DL. The released interleukin 2 receptor binds interleukin 2 efficiently. *J Immunol* 1986; **137**: 3841-3844.
12. Langholz E, Munkholm P, Nielsen OH, Kreiner S, Binder V. Incidence and prevalence of ulcerative colitis in the county of Copenhagen 1962 to 1987. *Scand J Gastroenterol* 1991; **26**: 1247-1256.
13. Binder V. A comparison between clinical state, macroscopic and microscopic appearances of rectal mucosa and cytologic picture of mucosal exudate in ulcertative colitis. *Scand J Gastroenterol* 1970; **5**: 627-632.
14. Uchiyama T, Nelson DL, Fleisher TA, Waldman TA. A monoclonal antibody (anti-Tac) reactive with activated and functionally mature human T-cells. II. Expression of Tac antigens on activated cytotoxic killer T cells, suppressor cells, and on one of two types of helper T cells. *J Immunol* 1981; **126**: 1398–1403.
15. Rubin LA, Kurman CC, Biddison WE, Goldman NE, Nelson DL. A monoclonal antibody, 7G7/B6, binds to an epitope on the human interleukin-2 receptor that is distinct from that recognized by interleukin-2 or anti-TAC. *Hybridoma* 1985; **4**: 91–102.

16. Hakimi J, Seals C, Anderson LE, *et al.* Biochemical and functional analysis of a soluble human interleukin-2 receptor produced in rodent cells. *J Biol Chem* 1987; **262:** 17336–17341.
17. Hafler DA, Fox DA, Manning ME, Schlossman SF, Reinherz EL, Weiner HL. *In vivo* activated T lymphocytes in the peripheral blood and cerebrospinal fluid of patients with multiple sclerosis. *N Engl J Med* 1983; **312:** 1405-1411.
18. Raedler A, Bredow G, Kirch W, Thiele HG, Greten H. *In vivo* activated T cells in autoimmune disease. *J Clin Lab Immunol* 1986; **19:** 181-186.
19. Youinou P, Le Groff P, Le Saout J, Courtois B, Shipley M, Lydyard PM. Activation of T lymphocytes in the blood and joints of patients with rheumatoid polyarthritis. *Rev Rhcum Mal Osteoartic* 1985; **52:** 473–477.
20. Duff GW. Peptide regulatory factors in non-malignant disease. *Lancet* 1989; **i:** 1432–1435.
21. Wood NC, Symons JA, Duff GW. Serum interleukin-2 receptor in rheumatoid arthritis: a prognostic indicator of disease activity? *J Autoimmun* 1988; **1:** 353–361.
22. Colver CB, Symons JA, Duff GW. Soluble interleukin-2 receptor in atopic eczema. *Br Med J* 1989; **298:** 1426-1428.
23. Symons JA, Wood NC, Di Giovine FS, Duff GW. Soluble interleukin-2 receptor in rheumatoid arthritis: correlation with disease activity, interleukin-1 and interleukin-2 inhibition. *J Immunol* 1988; **141:** 2612–2618.
24. Wolf RE, Brelsford WG. Soluble interleukin-2 receptors in systemic lupus erythematosus. *Arthritis Rheum* 1988; **31:** 729–735.
25. Lobo-Yeo A, Mieli-Verganis G, Mowat AP, Verganis D. Soluble interleukin 2 receptors in autoimmune chronic active hepatitis. *Gut* 1990; **31:** 690–693.
26. Fais S, Pallone F, Squarcia O, Biancone L, Ricci F, Paoluzzi P, Boirivant M. HLA-DR antigens on colonic epithelial cells in inflammatory bowel disease: I. Relation to the state of activation of lamina propria lymphocytes and to the epithelial expression of other surface markers. *Clin Exp Immunol* 1987; **68:** 605–612.
27. Pallone F, Fais S, Squarcia O, Biancone L, Pozzilli P, Boirivant M. Activation of peripheral blood and intestinal lamina propria lymphocytes in Crohn's disease. Gut 1990; **28:** 745-753.
28. Mueller C, Knoflach P, Zielinski CC. T-cell activation in Crohn's disease: Increased levels of soluble interleukin-2 receptor in serum and in supernatants of stimulated peripheral blood mononuclear cells. *Gastroenterology* 1990; **98:** 639-646.
29. Mahida YR, Gallagher A, Kurlac L, Hawkey CJ. Circulating and tissue interleukin 2 receptor levels in inflammatory bowel disease. *Clin Exp Immunol* 1990; **82:** 75–80.
30. Crabtree JE, Juby LD, Heatley RV, Lobo AJ, Bullimore DW, Axon ATR. Soluble interleukin-2 receptor in Crohn's disease: relation of serum concentrations to disease activity. *Gut* 1990; **31:** 1033-1036.
31. Choy MY, Walker-Smith JA, Williams CB, MacDonald TT. Differential expression of CD25 (interleukin-2 receptor) on lamina propria T cells and macrophages in the intestinal lesions in Crohn's disease and ulcerative colitis. *Gut* 1990; **31:** 1365-1370.
32. Matsuura T, West GA, Klein JS, Ferraris L, Fiocchi C. Soluble interleukin 2 and CD8 and CD4 receptors in inflammatory bowel disease. *Gastroenterology* 1992; **102:** 2006–2014.
33. Schreiber S, MacDermott RP, Raedler A, Pinnau R, Bertovich MJ, Nash GS. Increased activation of isolated intestinal lamina propria mononuclear cells in inflammatory bowel disease. *Gastroenterology* 1991; 1020–1030.
34. Schreiber S, MacDermott RP, Raedler A, Conn AF, Rombeau JL. Increased *in vitro* release of soluble interleukin-2 receptor by colonic lamina propria mononuclear cell in inflammatory bowel disease. *Gut* 1992; **33:** 734-742.
35. Jewell DP. Corticosteroids for the management of ulcerative colitis and Crohn's disease. *Gastroenterol Clin North Am* 1989; **18:** 21–34.
36. Tvede N, Brynskov J. Interleukin-2 stimulation of blood mononuclear cells from patients with inflammatory bowel disease. *APMIS* 1991; **99:** 759–764.
37. Degiannis D, Czarnecki M, Hornung N, Raskova J, Raska K. Mixed lymphocyte reaction-induced release of soluble IL-2 receptor. *Transplantation* 1991; **51:** 518–523.

ACKNOWLEDGEMENTS. The authors are grateful to Birgit Dejbjerg for her technical assistance. This study was supported by a grant from handelsgartner Ove Villiam Buhl Olesen and hustru Edith Buhl Olesen's Foundation, and Else and Mogens Wedell-Wedellsborg's Foundation.

**Received 27 November 1992; accepted 31 December 1992**
